# Dose–response relationship between physical activity and mental health outcomes in adolescents

**DOI:** 10.3389/fpubh.2026.1780794

**Published:** 2026-05-25

**Authors:** Yang Bai, Di Liang, Yan Lu, Weijun Zhang, Jiayan Huang, Yuxia Wu

**Affiliations:** 1 School of Public Health, NHC Key Laboratory of Health Technology Assessment, Fudan University, Shanghai, China; 2 Department of Education and Training, Shanghai Jiading Mental Health Center, Shanghai, China; 3 Department of Medicine, David Geffen School of Medicine, University of California Los Angeles, Los Angeles, CA, United States; 4 Yichuan Community Health Service Center of Putuo District, Shanghai, China

**Keywords:** adolescents, mental health, physical activity, psychological resilience, self-efficacy, stress perception

## Abstract

**Background:**

Physical activity is generally associated with improved adolescent mental health, yet its dose–response relationship remains unclear. This study examined how physical activity relates to stress perception, psychological resilience, and self-efficacy among adolescents.

**Methods:**

A cross-sectional survey was conducted in September 2024 among 2,270 students aged 12–15 years from 4 middle schools in Shanghai, China. Multivariable linear regression and restricted cubic spline (RCS) models were used to examine association and dose–response relationships, adjusting for demographic and academic covariates.

**Results:**

Higher physical activity was significantly associated with lower stress perception (*B* = −0.13, *p* < 0.001), higher psychological resilience (*B* = 0.33, *p* < 0.001), and higher self-efficacy (*B* = 0.25, *p* < 0.001). RCS analyses revealed dose–response relationships. At lower physical activity levels, the slopes were −5.65 for stress perception, 8.59 for psychological resilience, and 12.10 for self-efficacy (all *p* < 0.001), while at higher physical activity levels, the associations were substantially attenuated. Gender-stratified analyses indicated stronger associations in males than in females.

**Conclusion:**

Physical activity is associated with better mental health in adolescents, particularly among those with lower activity levels. The findings highlight the importance of targeting less active adolescents in school-based interventions.

## 1 Introduction

Mental health disorders among adolescents are a significant health issue, with an estimated global prevalence of approximately 15% (1, 2). In East Asia and the Pacific, the burden is particularly pronounced, largely driven by intense academic pressure (3). In China, the prevalence of mental health disorders among adolescents is estimated at 17.5% (4).

Evidence consistently shows that physical activity is associated with reduced depressive and anxiety symptoms in adolescents (5–9). However, fewer studies have investigated the relationship between physical activity and non-clinical mental health outcomes, such as stress perception, self-efficacy, and psychological resilience. Although a recent review identified 30 studies examining physical activity and mental health in children and adolescents, only seven specifically assessed stress perception as an outcome (9). Evidence for resilience and self-efficacy remains sparse and inconclusive (7). Notably, compared to adult populations, adolescent-focused research remains limited by small sample sizes and methodological weaknesses (6). Moreover, the existing evidence is geographically concentrated in high-income countries, with few studies conducted in China (9). Most studies demonstrate a positive relationship between activity levels and mental health outcomes; however, emerging evidence suggests that this association may be nonlinear, with diminishing returns or potential adverse outcomes, such as overuse injuries and psychological burnout, at higher activity levels (10, 11).

Recognizing the importance of physical activity, global and national guidelines have been developed to promote adolescent well-being. The World Health Organization (WHO) recommends at least 60 min of moderate-to-vigorous physical activity daily for adolescents (12). China has adopted this standard and, through the 2022 Compulsory Education Physical Education and Health Curriculum Standards, has further integrated physical activity with health education to promote student well-being (13–15). While these policies aim to raise baseline activity levels, the degree to which mental health benefits vary across the activity spectrum remains unclear. This highlights the need to explore the potential dose–response relationship.

This study focuses on the association between physical activity and three adolescent mental-health outcomes: stress perception, psychological resilience, and self-efficacy. In this study, stress perception refers to an individual’s subjective evaluation of external demands and their ability to cope (16). Psychological resilience is the ability to adapt positively in the face of stressors (17). Similarly, self-efficacy reflects confidence in one’s capacity to complete tasks (18). Both psychological resilience and self-efficacy act as protective factors that help adolescents manage stress (19).

To address existing knowledge gaps, we conducted a cross-sectional survey among middle school students in Shanghai, China, to examine the dose–response relationship between physical activity and key mental health indicators. The findings aim to provide empirical evidence to inform adolescent physical activity guidelines and support the development of school-based mental health promotion strategies.

## 2 Methods

### 2.1 Study design and sample

A cross-sectional, school-based survey was conducted in Shanghai, China, a major city actively implementing the 2022 Compulsory Education Physical Education and Health Curriculum Standards. Convenience sampling was used to select study sites. To account for regional economic differences, two districts were selected: Putuo (urban) and Jiading (rural). Within each district, two public nine-year integrated schools (grades 1–9) were selected. All students in grades 6–9 (aged 12–15) were invited to participate in the study. Due to scheduling constraints, ninth-grade classes in one school were excluded from the analysis.

The survey was conducted in September 2024. Informed consent was obtained from both parents and students. The electronic questionnaire was administered under the supervision of trained teachers. A total of 2,290 questionnaires were collected. After excluding 5 duplicate and 15 invalid responses, 2,270 valid questionnaires remained, resulting in a validity response rate of 99.1%. The valid responses covered 98.3% of the total student population (2,310 students). Specifically, the sample consisted of 941 students from School 1 (41.5%), 622 from School 2 (27.4%), 500 from School 3 (22.0%), and 207 from School 4 (9.1%).

### 2.2 Measurement instruments

#### 2.2.1 Outcome variables

Stress perception was measured by the Perceived Stress Scale (PSS) (20). The Chinese version (CPSS), translated by Yang and Huang (21) in 2003, consisted of 14 items. It assessed tension and loss of control using a 5-point Likert scale. Total scores ranged from 14 to 70, with higher scores indicating greater stress perception. CPSS scores were divided into three levels based on equal intervals of the total score range (low: 14–32; moderate: 33–51; high: 52–70), consistent with previous studies (22, 23). The Chinese version indicated acceptable reliability (Cronbach’s alpha = 0.808) and good validity in Chinese adolescents (24).

Psychological resilience was measured by the Connor-Davidson Resilience Scale (CD-RISC-10), rated on a 5-point Likert scale, with total scores ranging from 0 to 40 (25). Higher scores reflected greater resilience. The Chinese version, translated and adapted by Wang et al. (26), was widely used in China. It indicated excellent reliability (Cronbach’s alpha = 0.928) and strong psychometric properties in Chinese adolescents (27, 28).

Self-efficacy was assessed by the Rating of Emotion Regulation Self-Efficacy Scale (RESE), developed by Caprara et al. (29, 30) and revised by Wang and Dou. This scale used a 5-point Likert scale, with higher scores indicating greater confidence in emotion regulation. Total scores ranged from 18 to 90. The RESE revealed excellent reliability (Cronbach’s alpha = 0.944) and strong psychometric properties in Chinese adolescents (30).

#### 2.2.2 Primary predictor

The Physical Activity Questionnaire for Adolescents (PAQ-A), developed by the University of Saskatchewan, showed good internal consistency and construct validity in Canadian children and adolescents (31). The PAQ-A effectively differentiated between gender and age, providing a composite assessment of overall physical activity and moderate-to-vigorous physical activity over the past 7 days (32).

The Chinese version of the PAQ-A indicated acceptable reliability and validity. It was suitable for monitoring physical activity levels in large samples of Chinese adolescents (33). The PAQ-A used a 5-point scale, with higher scores indicating more physical activity. In this study, the PAQ-A showed strong internal consistency, with a Cronbach’s alpha of 0.889.

#### 2.2.3 Control variables

Demographic covariates included gender, age, grade level, family composition, only-child status, parental educational attainment, and academic satisfaction. Academic satisfaction was assessed using a single-item scale, where participants rated their satisfaction with academic performance on a 10-point continuum (1 = extremely dissatisfied, 10 = extremely satisfied). These variables were included as covariates in regression models to control for potential confounding effects on the primary outcomes.

### 2.3 Statistical analysis

#### 2.3.1 Descriptive statistics

Descriptive statistics included means with standard deviations for continuous variables and frequencies with percentages for categorical variables. Physical activity was classified into four ordered levels corresponding to quartiles of the total PAQ-A score: Q1 (1–2.07), Q2 (2.07–2.37), Q3 (2.37–2.77), and Q4 (2.77–5). Group comparisons for continuous variables were performed using Welch’s *t*-tests or one-way ANOVA as appropriate. For categorical variables, Fisher’s exact test was applied when any expected cell count was <5; otherwise, Chi-square tests were used.

#### 2.3.2 Multiple linear regression analysis

Multiple linear regression was performed to examine the relationships between physical activity and stress perception, psychological resilience, and self-efficacy. Covariates included gender, age, grade level, family composition, only-child status, parental educational attainment, and academic satisfaction. Two models were fitted. Model 1 examined the main effects of physical activity and covariates, while Model 2 additionally included an interaction term between physical activity and gender to assess potential effect modification by gender. Prior to regression analysis, physical activity, stress perception, psychological resilience, self-efficacy, and academic satisfaction were standardized using *Z*-scores. In addition, sensitivity analysis was conducted by replacing the continuous physical activity variable with its categorization into quartiles to assess the robustness of the results.

#### 2.3.3 Nonlinear regression analysis

Nonlinear associations were assessed using Restricted Cubic Spline (RCS) models for both the total sample and gender-stratified groups, considering potential gender differences. RCS functions were useful for examining dose–response relationships and were well-documented in the literature (34–36). The model generated curves with physical activity scores on the *x*-axis and standardized regression coefficients on the *y*-axis, with the inflection point at 0 (37, 38). Fixed knots were placed at the 25th, 50th, and 75th percentiles of physical activity (*k* = 3), and all models were adjusted for the covariates.

Nonlinear trends were tested based on the likelihood ratio test (*p*-for nonlinearity). Threshold effects, such as inflection or saturation points, were further examined using segmented regression. The likelihood ratio test was used to assess whether the segmented model provided a significantly better fit than the linear model by introducing an inflection point.

All statistical analyses were performed using the R software (version 4.2.2).

## 3 Results

### 3.1 Characteristics of study participants

A total of 2,270 adolescents were included in the analysis, comprising 1,222 males (53.8%) and 1,048 females (46.2%). The mean age of the sample was 12.8 years, and 84.8% of participants held local household registration. Physical activity was categorized into four quartiles (Q1–Q4). The proportion of males was higher in Q4 (61.8%) compared to Q1 (47.5%), whereas the proportion of females was lower in Q4 (38.2%) compared to Q1 (52.5%). These differences in sex distribution across quartiles were statistically significant (*p* < 0.001). In addition, academic satisfaction was higher at 7.63 in Q4 compared with 6.53 in Q1 (*p* < 0.001) (Table 1).

**Table 1:** Characteristics of participants by physical activity.

Variables	Physical activity^a^					*p*-value^b^
	Overall *N* = 2,270	Q1 [1, 2.07) *N* = 554	Q2 [2.07, 2.37) *N* = 519	Q3 [2.37, 2.77) *N* = 619	Q4 [2.77, 5] *N* = 578	
Stress perception	38.05 ± 8.60	41.17 ± 8.38	37.92 ± 7.45	37.29 ± 8.45	35.99 ± 9.13	<0.001
Stress perception level						<0.001
Low (<33)	560 (24.7%)	72 (13.0%)	115 (22.2%)	173 (27.9%)	200 (34.6%)	
Moderate (33–51)	1,582 (69.7%)	427 (77.1%)	385 (74.2%)	415 (67.0%)	355 (61.4%)	
High (≥52)	128 (5.6%)	55 (9.9%)	19 (3.7%)	31 (5.0%)	23 (4.0%)	
Psychological resilience	27.50 ± 9.14	22.28 ± 9.14	26.52 ± 8.38	29.00 ± 8.16	31.78 ± 8.15	<0.001
Self-efficacy	65.72 ± 14.08	59.15 ± 13.74	65.32 ± 12.88	66.87 ± 13.74	71.16 ± 13.23	<0.001
Academic satisfaction	7.13 ± 2.32	6.53 ± 2.50	6.98 ± 2.08	7.31 ± 2.18	7.63 ± 2.34	<0.001
Age	12.81 ± 1.19	12.86 ± 1.20	12.77 ± 1.21	12.84 ± 1.17	12.75 ± 1.17	0.339
Gender						<0.001
Male	1,222 (53.8%)	263 (47.5%)	256 (49.3%)	346 (55.9%)	357 (61.8%)	
Female	1,048 (46.2%)	291 (52.5%)	263 (50.7%)	273 (44.1%)	221 (38.2%)	
Grade						0.032
6th grade	750 (33.0%)	171 (30.9%)	169 (32.6%)	199 (32.1%)	211 (36.5%)	
7th grade	617 (27.2%)	141 (25.5%)	149 (28.7%)	169 (27.3%)	158 (27.3%)	
8th grade	548 (24.1%)	158 (28.5%)	131 (25.2%)	150 (24.2%)	109 (18.9%)	
9th grade	355 (15.6%)	84 (15.2%)	70 (13.5%)	101 (16.3%)	100 (17.3%)	
Household registration						0.027
Local household registration	1,927 (84.9%)	483 (87.2%)	445 (85.7%)	530 (85.6%)	469 (81.1%)	
Non-local household registration	343 (15.1%)	71 (12.8%)	74 (14.3%)	89 (14.4%)	109 (18.9%)	
Only child						0.586
Yes	1,472 (64.8%)	370 (66.8%)	341 (65.7%)	393 (63.5%)	368 (63.7%)	
No	798 (35.2%)	184 (33.2%)	178 (34.3%)	226 (36.5%)	210 (36.3%)	
Mother’s education level						0.013
High school or below	383 (16.9%)	104 (18.8%)	86 (16.6%)	96 (15.5%)	97 (16.8%)	
Associate/undergraduate degree	1,109 (48.9%)	254 (45.8%)	250 (48.2%)	315 (50.9%)	290 (50.2%)	
Master’s degree or above	243 (10.7%)	42 (7.6%)	53 (10.2%)	73 (11.8%)	75 (13.0%)	
Not sure	535 (23.6%)	154 (27.8%)	130 (25.0%)	135 (21.8%)	116 (20.1%)	
Father’s education level						<0.001
High school or below	334 (14.7%)	96 (17.3%)	65 (12.5%)	87 (14.1%)	86 (14.9%)	
Associate/undergraduate degree	1,040 (45.8%)	239 (43.1%)	235 (45.3%)	283 (45.7%)	283 (49.0%)	
Master’s degree or above	324 (14.3%)	50 (9.0%)	78 (15.0%)	92 (14.9%)	104 (18.0%)	
Not sure	572 (25.2%)	169 (30.5%)	141 (27.2%)	157 (25.4%)	105 (18.2%)	

a Mean ± SD; *n* (%).

b One-way analysis of means; Pearson’s Chi-squared test.

Overall, the sample reported a moderate level of stress perception (mean CPSS score: 38.05/70). Based on this classification, 24.7% of adolescents were categorized as having low stress, 69.7% moderate stress, and 5.6% high stress. The mean psychological resilience score was 27.50/40 (CD-RISC-10), comparable to values reported for Chinese adolescents (39). Self-efficacy averaged 65.72/90 (RESE), above the theoretical midpoint of the scale (45/90), suggesting a moderate-to-high level of self-efficacy (Table 1).

### 3.2 Association between physical activity and mental health outcomes

Higher physical activity levels were associated with more favorable mental health outcomes. Adolescents in the highest physical activity group had a mean stress perception score of 35.99/70, compared with 41.17/70 in the lowest group (*p* < 0.001). Mean psychological resilience was 31.78/40 versus 22.28/40 in the lowest group (*p* < 0.001), and mean self-efficacy was 71.16/90 compared with 59.15/90 (*p* < 0.001) (Table 1).

In regression analyses, higher levels of physical activity were associated with better mental health outcomes in adolescents. Physical activity was inversely related to stress perception (*B* = −0.13, *p* < 0.001), indicating that a 1-standard deviation higher physical activity score was associated with a 0.13-standard deviation lower stress perception score. Positive associations were observed with psychological resilience (*B* = 0.33, *p* < 0.001) and self-efficacy (*B* = 0.25, *p* < 0.001). In the moderation model, the interaction between physical activity and gender was significant for self-efficacy (*B* = 0.10, *p* = 0.015), while no significant interaction was observed for stress perception or psychological resilience (Table 2).

**Table 2:** Linear regression analysis of the association between physical activity and stress perception, psychological resilience, and self-efficacy^a^.

Variables	Stress perception				Psychological resilience				Self-efficacy			
	Model 1 *β* (95% CI)	*p*-value	Model 2 *β* (95% CI)	*p*-value	Model 1 *β* (95% CI)	*p*-value	Model 2 *β* (95% CI)	*p*-value	Model 1 *β* (95% CI)	*p*-value	Model 2 *β* (95% CI)	*p*-value
Physical activity	−0.13 (−0.17, −0.09)	<0.001	−0.15 (−0.21, −0.09)	<0.001	0.33 (0.29, 0.36)	<0.001	0.30 (0.24, 0.35)	<0.001	0.25 (0.21, 0.29)	<0.001	0.19 (0.13, 0.25)	<0.001
Gender × physical activity	—	—	0.03 (−0.05, 0.10)	0.520	—	—	0.05 (−0.02, 0.12)	0.192	—	—	0.10 (0.02, 0.17)	0.015
Academic satisfaction	−0.30 (−0.34, −0.26)	<0.001	−0.30 (−0.34, −0.26)	<0.001	0.29 (0.25, 0.32)	<0.001	0.29 (0.25, 0.32)	<0.001	0.25 (0.21, 0.29)	<0.001	0.25 (0.21, 0.29)	<0.001
Age	−0.05 (−0.12, 0.02)	0.143	−0.05 (−0.12, 0.02)	0.141	0.01 (−0.05, 0.07)	0.783	0.01 (−0.06, 0.07)	0.797	0.00 (−0.07, 0.07)	0.967	0.00 (−0.07, 0.07)	0.994
Gender (vs. female)												
Male	−0.01 (−0.09, 0.07)	0.788	−0.01 (−0.09, 0.07)	0.810	0.08 (0.01, 0.16)	0.022	0.09 (0.01, 0.16)	0.019	0.08 (0.00, 0.15)	0.048	0.08 (0.00, 0.16)	0.038
Grade (vs. 6th grade)												
7th grade	0.25 (0.13, 0.37)	<0.001	0.25 (0.13, 0.37)	<0.001	−0.17 (−0.28, −0.07)	0.002	−0.17 (−0.28, −0.07)	0.002	−0.23 (−0.35, −0.12)	<0.001	−0.23 (−0.35, −0.12)	<0.001
8th grade	0.35 (0.18, 0.52)	<0.001	0.35 (0.18, 0.52)	<0.001	−0.20 (−0.36, −0.05)	0.012	−0.20 (−0.36, −0.05)	0.011	−0.20 (−0.37, −0.03)	0.018	−0.20 (−0.37, −0.04)	0.018
9th grade	0.11 (−0.12, 0.34)	0.352	0.11 (−0.12, 0.34)	0.349	0.12 (−0.10, 0.33)	0.297	0.12 (−0.10, 0.33)	0.292	0.14 (−0.08, 0.37)	0.215	0.15 (−0.08, 0.37)	0.207
Household registration (vs. non-local household registration)												
Local household registration	−0.05 (−0.16, 0.06)	0.395	−0.05 (−0.16, 0.06)	0.385	−0.07 (−0.17, 0.04)	0.198	−0.07 (−0.17, 0.03)	0.186	−0.05 (−0.16, 0.06)	0.363	−0.05 (−0.16, 0.05)	0.328
Only child (vs. no)												
Yes	−0.10 (−0.18, −0.01)	0.021	−0.10 (−0.18, −0.02)	0.020	0.10 (0.03, 0.18)	0.008	0.10 (0.03, 0.18)	0.008	0.12 (0.04, 0.20)	0.004	0.12 (0.04, 0.20)	0.004
Mother’s education level (vs. high school or below)												
Associate/undergraduate degree	−0.10 (−0.22, 0.02)	0.109	−0.10 (−0.22, 0.02)	0.108	0.06 (−0.05, 0.17)	0.319	0.06 (−0.05, 0.16)	0.325	0.08 (−0.03, 0.20)	0.165	0.08 (−0.04, 0.20)	0.172
Master’s degree or above	0.08 (−0.09, 0.26)	0.351	0.08 (−0.09, 0.26)	0.358	0.02 (−0.14, 0.19)	0.808	0.02 (−0.15, 0.18)	0.828	−0.03 (−0.20, 0.15)	0.747	−0.03 (−0.21, 0.14)	0.712
Not sure	−0.04 (−0.20, 0.12)	0.616	−0.04 (−0.20, 0.12)	0.597	0.08 (−0.07, 0.22)	0.318	0.07 (−0.08, 0.22)	0.347	0.06 (−0.10, 0.21)	0.457	0.05 (−0.11, 0.21)	0.524
Father’s education level (vs. high school or below)												
Associate/undergraduate degree	−0.03 (−0.16, 0.09)	0.615	−0.03 (−0.16, 0.09)	0.613	0.08 (−0.04, 0.19)	0.199	0.08 (−0.04, 0.19)	0.201	0.02 (−0.10, 0.14)	0.726	0.02 (−0.10, 0.14)	0.734
Master’s degree or above	−0.05 (−0.22, 0.12)	0.559	−0.05 (−0.22, 0.12)	0.555	0.12 (−0.03, 0.28)	0.122	0.12 (−0.03, 0.28)	0.125	0.00 (−0.17, 0.16)	0.974	0.00 (−0.17, 0.16)	0.955
Not sure	0.04 (−0.12, 0.20)	0.633	0.04 (−0.12, 0.20)	0.624	−0.09 (−0.24, 0.06)	0.234	−0.09 (−0.24, 0.06)	0.245	−0.06 (−0.21, 0.10)	0.472	−0.05 (−0.21, 0.10)	0.502

^a^Model 1: adjusted for age, gender, grade, household registration, only child status, parental education level, and academic satisfaction. Model 2: model 1 additionally included an interaction term between physical activity and gender.

### 3.3 Dose–response relationship between physical activity and mental health outcomes

#### 3.3.1 Stress perception

**Figure 1:**
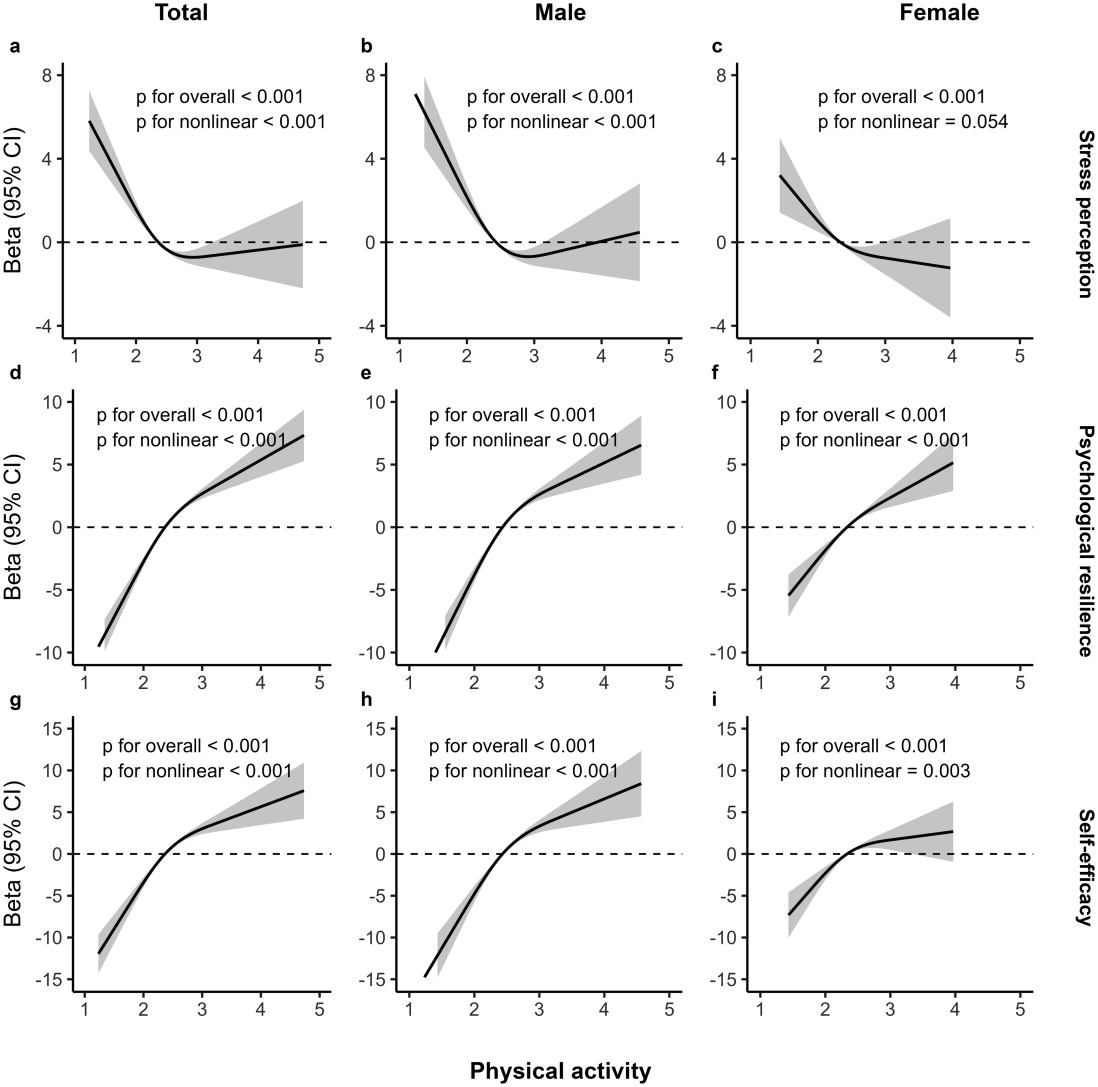
Cubic spline models showing the relationship between physical activity and stress perception, psychological resilience, and self−efficacy across gender subgroups. Plots **(a,d,g)** show the results of the restricted cubic spline regression model analysis for stress perception, psychological resilience, and self-efficacy, respectively. The figure demonstrates the associations between independent variables and these outcomes, adjusted for covariates including age, gender, grade, household registration, parental education level, academic satisfaction, and only-child status. The *y*-axis represents beta values, reflecting the difference in stress perception, psychological resilience, and self-efficacy for any given level of physical activity compared to the reference value (50th percentile). Shaded areas denote 95% confidence intervals (CIs). Plots **(b,e,h)** and **(c,f,i)** display the results for male and female subgroups, respectively.

Stress perception was inversely associated with higher physical activity, with a more pronounced association below a PAQ-A score of 2.33 (*p*-for nonlinearity < 0.001) (Figure 1a). Each one-point higher activity level was associated with a 5.65-point lower stress perception score (*B* = −5.65, *p* < 0.001). Above this threshold, the effect was minimal (*B* = −0.23, *p* = 0.636). The segmented model fit better than the linear model (*p* < 0.001) (Table 3).

**Table 3:** Threshold effect analysis of physical activity on mental health outcomes.

Outcome	Model^a^	Inflection point	Effect (95% CI)	*p*-value	*p* for LRT^b^
Stress perception	Model 1	<2.33	−5.65 (−7.44, −3.87)	<0.001	<0.001
		≥2.33	−0.23 (−1.18, 0.72)	0.636	
	Model 2	<2.30	−7.33 (−9.92, −4.74)	<0.001	<0.001
		≥2.30	−0.09 (−1.21, 1.03)	0.872	
	Model 3^c^	—	—	—	—
		—	—	—	
Psychological resilience	Model 1	<2.40	8.59 (6.93, 10.26)	<0.001	<0.001
		≥2.40	3.12 (2.15, 4.09)	<0.001	
	Model 2	<2.07	14.44 (10.45, 18.43)	<0.001	<0.001
		≥2.07	3.94 (2.97, 4.90)	<0.001	
	Model 3	<2.60	5.69 (3.87, 7.52)	<0.001	0.075
		≥2.60	2.49 (0.35, 4.62)	0.022	
Self-efficacy	Model 1	<2.27	12.10 (8.75, 15.45)	<0.001	<0.001
		≥2.27	3.61 (2.20, 5.02)	<0.001	
	Model 2	<2.17	16.47 (10.99, 21.95)	<0.001	<0.001
		≥2.17	4.66 (2.96, 6.36)	<0.001	
	Model 3	<2.97	6.14 (4.07, 8.20)	<0.001	0.008
		≥2.97	−2.22 (−8.06, 3.61)	0.455	

a Model 1 represents the overall sample model. Model 2 and Model 3 represent subgroup models for males and females.

b LRT, likelihood ratio test.

c For stress perception in females, nonlinearity was not statistically significant (*p* = 0.054); therefore, threshold effect analysis was not conducted (—indicates not applicable).

In gender-stratified analyses, a similar nonlinear pattern was observed in males, with a threshold at 2.30, beyond which the effect remained stable (*p*-for nonlinearity <0.001) (Figure 1b; Table 3). In females, nonlinearity was not significant (*p* = 0.054) (Figure 1c).

#### 3.3.2 Psychological resilience

Physical activity showed a stronger correlation with psychological resilience at lower activity levels, as reflected by the steeper slope (*p*-for nonlinearity <0.001) (Figure 1d). Resilience scores were higher with increasing physical activity below a PAQ-A score of 2.40, with each one-point higher activity level associated with an 8.59-point higher resilience score (*B* = 8.59, *p* < 0.001). Above this threshold, the slope flattened, with each additional point in activity associated with a 3.12-point higher resilience score (*B* = 3.12, *p* < 0.001) (Table 3).

In gender-stratified analyses, males showed a stronger effect, with a *B* value of 11.65 below the threshold of 2.07 (*p* < 0.001) and 3.94 above it (*p* < 0.001). In females, the association was weaker, with a *B* value of 5.69 below the threshold of 2.60 (*p* < 0.001) and 2.49 above it (*p* = 0.022) (Figures 1e,f; Table 3).

#### 3.3.3 Self-efficacy

The association between physical activity and self-efficacy showed a pattern comparable to that for psychological resilience (Figure 1g). Below a PAQ-A score of 2.27, each one-point higher activity level was associated with a 12.10-point higher self-efficacy score (*B* = 12.10, *p* < 0.001). Above this threshold, the association weakened, with each additional point in activity associated with a 3.61-point higher self-efficacy score (*B* = 3.61, *p* < 0.001) (Table 3).

In males, physical activity showed a stronger effect on self-efficacy than in females (Figures 1h,i). The *B* value was 16.47 below the threshold of 2.17 (*p* < 0.001) and 4.66 above it (*p* < 0.001). In females, the association was weaker (*B* = 6.14 below the threshold of 2.97, *p* < 0.001; non-significant above it, *p* = 0.455) (Table 3).

### 3.4 Sensitivity analysis

Sensitivity analyses categorizing physical activity into quartiles were conducted to examine the robustness of the results. The analysis showed consistent effects across all mental health outcomes, aligning with the trends observed in the multivariable regression using physical activity as a continuous variable. Adolescents in the highest quartile had significantly better outcomes in stress perception (*B* = −3.64), psychological resilience (*B* = 7.51), and self-efficacy (*B* = 9.59), with all results reaching statistical significance (*p* < 0.001). In the interaction models, gender significantly moderated the association between physical activity quartiles and self-efficacy (Q4 × male: *B* = 4.53, *p* = 0.003) and psychological resilience (Q4 × male: *B* = 2.11, *p* = 0.027), whereas no significant interaction was observed for stress perception (Table 4).

**Table 4:** Associations of physical activity quartiles with stress perception, psychological resilience, and self-efficacy^a^.

Variables	Stress perception				Psychological resilience				Self-efficacy			
	Model 1 *β* (95% CI)	*p*-value	Model 2 *β* (95% CI)	*p*-value	Model 1 *β* (95% CI)	*p*-value	Model 2 *β* (95% CI)	*p*-value	Model 1 *β* (95% CI)	*p*-value	Model 2 *β* (95% CI)	*p*-value
Physical activity (vs. Q1)												
Q2 [2.07, 2.37)	−2.72 (−3.64, −1.79)	<0.001	−1.94 (−3.23, −0.64)	0.003	3.46 (2.54, 4.38)	<0.001	2.82 (1.53, 4.10)	<0.001	5.28 (3.79, 6.77)	<0.001	3.77 (1.69, 5.85)	<0.001
Q3 [2.37, 2.77)	−3.05 (−3.96, −2.14)	<0.001	−3.14 (−3.86, −2.03)	<0.001	5.62 (4.71, 6.52)	<0.001	4.90 (3.60, 6.20)	<0.001	6.50 (5.03, 7.96)	<0.001	5.48 (3.37, 7.58)	<0.001
Q4 [2.77, 5]	−3.64 (−4.58, −2.69)	<0.001	−3.76 (−4.45, −1.83)	<0.001	7.51 (6.57, 8.46)	<0.001	6.38 (4.97, 7.78)	<0.001	9.59 (8.06, 11.11)	<0.001	7.12 (4.85, 9.38)	<0.001
Gender × physical activity (vs. Q1 × gender)												
Q2 × male	—	—	−1.59 (−3.43, 0.25)	0.091	—	—	1.34 (−0.50, 3.17)	0.154	—	—	3.13 (0.17, 6.10)	0.038
Q3 × male	—	—	0.07 (−1.74, 1.88)	0.940	—	—	1.46 (−0.34, 3.26)	0.112	—	—	2.18 (−0.73, 5.08)	0.142
Q4 × male	—	—	−1.07 (−2.94, 0.81)	0.266	—	—	2.11 (0.24, 3.97)	0.027	—	—	4.53 (1.51, 7.54)	0.003
Academic satisfaction	−1.09 (−1.24, −0.94)	<0.001	−1.10 (−1.24, −0.95)	<0.001	1.16 (1.01, 1.30)	<0.001	1.16 (1.01, 1.30)	<0.001	1.54 (1.31, 1.78)	<0.001	1.54 (1.31, 1.78)	<0.001
Age	−0.44 (−1.03, 0.15)	0.148	−0.42 (−1.01, 0.17)	0.161	0.06 (−0.53, 0.64)	0.850	0.04 (−0.54, 0.63)	0.881	0.00 (−0.95, 0.95)	0.994	−0.02 (−0.97, 0.92)	0.959
Gender (vs. female)												
Male	−0.12 (−0.78, 0.54)	0.717	0.49 (−0.78, 1.76)	0.450	0.90 (0.24, 1.56)	0.007	−0.28 (−1.55, 0.98)	0.661	1.22 (0.16, 2.29)	0.024	−1.14 (−3.18, 0.91)	0.277
Grade (vs. 6th grade)												
7th grade	2.21 (1.20, 3.21)	<0.001	2.17 (1.17, 3.18)	<0.001	−1.69 (−2.69, −0.69)	<0.001	−1.69 (−2.70, −0.69)	<0.001	−3.43 (−5.05, −1.81)	<0.001	−3.42 (−5.04, −1.80)	<0.001
8th grade	3.06 (1.60, 4.52)	<0.001	3.03 (1.57, 4.49)	<0.001	−1.98 (−3.43, −0.53)	0.008	−1.99 (−3.44, −0.54)	0.007	−2.95 (−5.30, −0.61)	0.014	−2.95 (−5.30, −0.61)	0.013
9th grade	0.92 (−1.08, 2.92)	0.367	0.87 (−1.13, 2.87)	0.393	1.18 (−0.81, 3.17)	0.244	1.22 (−0.77, 3.21)	0.229	2.15 (−1.06, 5.37)	0.190	2.24 (−0.97, 5.46)	0.171
Household registration (vs. non-local household registration)												
Local household registration	−0.40 (−1.35, 0.55)	0.408	−0.40 (−1.35, 0.56)	0.414	−0.79 (−1.73, 0.16)	0.104	−0.81 (−1.76, 0.14)	0.095	−0.84 (−2.38, 0.69)	0.281	−0.89 (−2.42, 0.65)	0.257
Only child (vs. no)												
Yes	−0.83 (−1.53, −0.13)	0.020	−0.83 (−1.54, −0.13)	0.020	0.93 (0.23, 1.63)	0.009	0.93 (0.23, 1.63)	0.009	1.63 (0.50, 2.76)	0.005	1.62 (0.50, 2.75)	0.005
Mother’s education level (vs. High school or below)												
Associate/undergraduate degree	−0.86 (−1.85, 0.18)	0.105	−0.82 (−1.83, 0.20)	0.115	0.57 (−0.44, 1.58)	0.270	0.54 (−0.47, 1.55)	0.294	1.25 (−0.38, 2.89)	0.133	1.19 (−0.44, 2.82)	0.153
Master’s degree or above	0.67 (−0.86, 2.20)	0.393	0.67 (−0.86, 2.20)	0.392	0.34 (−1.18, 1.86)	0.660	0.32 (−1.21, 1.84)	0.684	−0.15 (−2.62, 2.31)	0.902	−0.20 (−2.66, 2.26)	0.872
Not sure	−0.35 (−1.71, 1.01)	0.613	−0.31 (−1.67, 1.05)	0.656	0.65 (−0.71, 2.00)	0.350	0.56 (−0.79, 1.92)	0.416	0.79 (−1.40, 2.99)	0.478	0.62 (−1.57, 2.81)	0.580
Father’s education level (vs. High school or below)												
Associate/undergraduate degree	−0.21 (−1.28, 0.87)	0.707	−0.18 (−1.26, 0.89)	0.738	0.67 (−0.40, 1.74)	0.220	0.67 (−0.40, 1.74)	0.223	0.21 (−1.52, 1.94)	0.812	0.18 (−1.55, 1.91)	0.835
Master’s degree or above	−0.30 (−1.74, 1.15)	0.685	−0.21 (−1.66, 1.23)	0.772	1.14 (−0.30, 2.58)	0.120	1.11 (−0.33, 2.55)	0.131	−0.17 (−2.49, 2.16)	0.889	−0.29 (−2.61, 2.04)	0.809
Not sure	0.43 (−0.94, 1.80)	0.537	0.46 (−0.92, 1.83)	0.515	−0.87 (−2.24, 0.50)	0.212	−0.84 (−2.20, 0.53)	0.230	−0.92 (−3.13, 1.29)	0.416	−0.88 (−3.09, 1.33)	0.434

^a^Model 1: Adjusted for age, gender, grade, household registration, only child status, parental education level, and academic satisfaction. Model 2: Model 1 additionally included an interaction term between physical activity and gender.

## 4 Discussion

This study demonstrates that higher levels of physical activity are associated with lower stress perception and higher psychological resilience and self-efficacy among adolescents, consistent with prior evidence (40–42). Importantly, the associations were most pronounced at lower levels of physical activity, indicating that even modest differences in activity levels among less active adolescents can yield meaningful mental health benefits. In addition, gender-stratified analyses revealed stronger associations in male adolescents than in females.

### 4.1 Stronger mental health benefits at lower physical activity levels

A key finding of this study is the identification of a stronger association between physical activity and mental health among adolescents with low physical activity levels. They are more likely to engage in sedentary and excessive screen time, both well-documented risk factors for poorer mental health (43). Furthermore, low activity is also linked to overweight and obesity, which can negatively affect body image, self-esteem, and overall psychological well-being (44, 45). Similar dose–response patterns have also been reported for other health outcomes, including all-cause mortality and several types of cancer, with the greatest risk reductions observed when individuals move from very low levels of physical activity to even modest activity levels (46, 47). Increasing physical activity in adolescents may help reduce multiple co-occurring risks and support better mental health outcomes.

Despite well-established global guidelines, adherence to recommended physical activity levels remains low. More than 80% of adolescents worldwide fail to meet the WHO recommendation of at least 60 min of moderate-to-vigorous physical activity per day, and only about 28.7% of Chinese adolescents achieve this standard (48, 49). Notably, previous research has estimated a PAQ-A cut-off of approximately 2.75 to discriminate adolescents meeting the 60 min daily moderate-to-vigorous physical activity (MVPA) recommendation (50). The inflection point identified in our study fell below this threshold, suggesting stronger associations among adolescents not meeting recommended activity levels. Our findings reinforce the importance of increasing physical activity among the least active adolescents, even if they do not yet meet the full recommended level.

### 4.2 Males exhibit stronger mental health benefits from physical activity than females

Our analysis reveals significant gender-based differences in the mental health effects of physical activity, with stronger associations observed in male adolescents. This finding aligns with previous studies (51, 52), and may be explained by behavioral and psychological factors. Males are more likely to participate in organized or competitive group sports (53), which fosters social connectedness and contributes positively to emotional well-being (54). These factors could promote positive self-cognition and mental health (55).

These observations underscore the need for gender-sensitive approaches to mental health promotion. Interventions should consider the different motivations, preferences, and barriers that male and female adolescents face when engaging in physical activity.

### 4.3 Physical activity shows stronger associations with adolescent mental health outcomes

An additional finding of this study is that physical activity showed a stronger connection to mental health outcomes than academic satisfaction. Previous research indicates that academic-related factors are also linked to adolescent mental health (56). However, the mechanisms through which academic satisfaction and physical activity relate to mental health appear to differ. Physical activity may influence emotional states through multiple neurobiological pathways, including increased brain-derived neurotrophic factor (BDNF), dopamine, and endorphin levels (57, 58). From the perspective of self-determination theory (SDT), physical activity may also support mental health by satisfying basic psychological needs for autonomy, competence, and relatedness, thereby strengthening self-efficacy and social connectedness (59).

In contrast, academic satisfaction represents a cognitive evaluation that is more strongly influenced by contextual factors such as academic pressure and social comparison (60). In highly competitive educational environments such as those in China, satisfaction with academic performance may still coexist with substantial academic pressure (61). Physical activity may affect emotional and stress-related processes through more direct biological and psychological pathways. Given these findings, it is essential for policymakers and educators to prioritize physical activity as a core component of adolescent mental health strategies.

## 5 Limitations

Several limitations should be considered. First, the findings may not be fully generalizable, as the sample was drawn from a single region in Shanghai, China, which may not represent the broader population. Second, the cross-sectional design limited the ability to draw causal inferences between physical activity and mental health outcomes. Third, self-reported physical activity data may introduce recall and reporting biases, as participants may overestimate their activity levels due to social desirability, potentially affecting the accuracy and reliability of the results. Therefore, future research should further explore the complex relationship between physical activity and mental health outcomes. Longitudinal studies with randomized sampling are needed to validate these findings.

## 6 Conclusion

This study provides evidence that higher levels of physical activity are associated with more favorable mental health outcomes among adolescents, including low stress perception and higher psychological resilience and self-efficacy. The observed dose–response relationship suggests that the greater benefits are seen in adolescents with lower levels of physical activity. These findings emphasize the importance of promoting physical activity not only as a means of improving physical health but also as a preventive strategy for mental health problems.

To maximize public health impact, policymakers and educators should prioritize the development of supportive environments and programs that encourage adolescent engagement in physical activity, especially among those who are least active. Tailored interventions that foster interest and remove barriers to participation are essential for improving adolescent well-being, a foundation for lifelong mental health.

## Data Availability

The raw data supporting the conclusions of this article will be made available by the authors, without undue reservation.
